# Scoring Protein Relationships in Functional Interaction Networks Predicted from Sequence Data

**DOI:** 10.1371/journal.pone.0018607

**Published:** 2011-04-19

**Authors:** Gaston K. Mazandu, Nicola J. Mulder

**Affiliations:** Computational Biology Group, Department of Clinical Laboratory Sciences, Institute of Infectious Disease and Molecular Medicine, University of Cape Town, Cape Town, South Africa; Baylor College of Medicine, United States of America

## Abstract

The abundance of diverse biological data from various sources constitutes a rich source of knowledge, which has the power to advance our understanding of organisms. This requires computational methods in order to integrate and exploit these data effectively and elucidate local and genome wide functional connections between protein pairs, thus enabling functional inferences for uncharacterized proteins. These biological data are primarily in the form of sequences, which determine functions, although functional properties of a protein can often be predicted from just the domains it contains. Thus, protein sequences and domains can be used to predict protein pair-wise functional relationships, and thus contribute to the function prediction process of uncharacterized proteins in order to ensure that knowledge is gained from sequencing efforts. In this work, we introduce information-theoretic based approaches to score protein-protein functional interaction pairs predicted from protein sequence similarity and conserved protein signature matches. The proposed schemes are effective for data-driven scoring of connections between protein pairs. We applied these schemes to the *Mycobacterium tuberculosis* proteome to produce a homology-based functional network of the organism with a high confidence and coverage. We use the network for predicting functions of uncharacterised proteins.

**Availability:**

Protein pair-wise functional relationship scores for *Mycobacterium tuberculosis* strain CDC1551 sequence data and python scripts to compute these scores are available at http://web.cbio.uct.ac.za/~gmazandu/scoringschemes.

## Introduction

In recent years we have experienced an exponential growth of biological data, including primary data such as genomic sequences resulting from worldwide DNA sequencing efforts and as well as functional data from high-throughput experiments, respectively. This abundance of primary sequence data and the large availability of public gene and protein sequence databases have the capability to provide many new insights into the biology of organisms. Several studies have shown that very often functional properties of a protein are not necessarily determined by the whole sequence but only by some of its sub-sequences [1]. Sequences sharing similar or conserved features are referred to as homologous sequences, and these features can be used for inferring and scoring protein pair-wise functional connections. One of these features is a protein domain, defined as a part of a protein sequence and structure that can evolve, function and exist independently of the rest of the protein chain [2].

Discovering sequence homology and modelling functional interactions between homologues from sequence and experimental data constitutes an important problem in molecular biology, as these can help to describe their behaviour in cellular processes and reveal the interplay between particular genes and proteins. In order to determine functional similarity between proteins, many approaches try to identify the sub-sequences of the proteins that may contribute to their function. Several Bioinformatics tools have been designed for deriving and storing these functional features. These include standard sequence comparison tools such as BLAST [3], [4], protein sequence databases such as UniProt [5], and protein signature databases such as InterPro [6], which integrates together predictive models or protein signatures representing protein domains, families and functional sites, from multiple source databases, namely, PROSITE, Pfam, PRINTS, ProDom, SMART, TIGRFAMs, PIRSF and SUPERFAMILY, Gene3D, PANTHER [7].

Using homologous datasets obtained from pair-wise sequence similarities, and protein domains and families in public databases, the inference of functional connections can be carried out based on the fact that two proteins sharing common domains or belonging to the same family are more likely to be functionally linked [8], 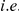, have similar functions with respect to molecular function and biological process. Note, the interactions discussed here are potential functional interactions, not direct physical interactions. These functional associations may be set in Boolean or binary form, 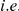, either two genes or proteins are functionally linked in which case the score is 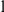 or they are not and the score is 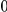. Such a scoring scheme is not consistent since it does not take into account the nature of parameters used to derive these functional associations. Understanding the properties of these functional relationships is key to successful mathematical modelling of such a system and developing efficient scoring techniques.

There are several problems with generating functional interaction networks using diverse data types such as sequence and functional genomics data. Considering that we are dealing with inaccurate data obtained from different experiments [9], [10], the uncertainty of data and noise inherent in each experiment must be efficiently managed by systematically weighing or scoring these functional associations [11]. This is referred to as a reliability or confidence score of functional associations for the particular computational approach used for prediction. This produces a graph with confidence-weighted relationships between each protein pair, which weighs each evidence type on the basis of its accuracy. Data-driven prediction methods should be able to extract essential features from particular datasets and to discount unwanted information. So, these scoring schemes must be data source and technology dependent, meaning that a given scoring scheme should normally vary according to the data sources and be designed on the basis of the technology used. Furthermore, the effectiveness of a scoring scheme for functional associations is critical for the quality of the analyses performed on the resulting network, including functional and structural analysis. An inability to accurately infer and score these protein pair functional associations leads to the propagation of annotation errors [12] and may negatively impact on the prediction analyses performed on the basis of these networks.

Several scoring schemes have been proposed for sequence data and are, so far, limited to only finding the similarity scores of proteins that are referred to as scoring functions. In the case of protein domain and family data, the scoring function is deduced from the number of common signatures shared by two proteins [10], [13]. These schemes miss other features related to the data under consideration including their nature and sources. On the other hand, for sequence similarity data this scoring function is just the 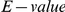 obtained from sequence comparison tools, and pair-wise functional interactions between proteins are obtained by simply applying an 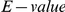 cut-off [10], [14]–[17]. However, there is no single fixed 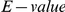 describing where homology ends and non homology begins. This shows that these schemes are not equipped to meet the requirements for scoring functional relationships, 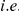, they do not capture all information shared between sequences.

In order to overcome these shortcomings, we propose an information-theoretic based measure to score protein-protein relationships in functional interaction networks predicted from homology data. This approach is shown to be effective for scoring functional pair-wise relationships from homology data, and translating the amount of biological content shared between proteins into the score of their functional relationships. We apply our method to score functional relationships between proteins in *Mycobacterium tuberculosis* (MTB) strain CDC1551 to produce a functional network from sequence data for this organism. This approach is compared to the STRING (Search Tool for the Retrieval of Interacting Genes/Proteins) [11], [18] homology scoring system for sequence similarity, and to existing scoring schemes for protein family and domain sharing [10], [13] in terms of functional classification coherence. Results show that the new scoring approach is as effective as that of the STRING approach, but produces a reliable functional network with higher coverage. The MTB functional network produced is then used to predict the functional class of proteins of unknown function, evaluated using leave-one-out cross validation.

## Materials and Methods

This section describes novel scoring schemes for protein family and domain data extracted from protein family databases, as well as for protein sequence similarity obtained by running sequence comparison tools such as Basic Local Alignment Search Tool (BLAST). Sequences in Fasta format and InterPro data for the organism were downloaded from the Integr8 project of the European Bioinformatics Institute (EBI) at http://www.ebi.ac.uk/integr8. Scoring functional relationships for data from protein families and domains has been widely addressed by the Bionformatics community. However, the approaches described so far in the literature are limited to finding the similarity scores between proteins by the number of common signatures shared by proteins. Two examples of such a scheme are given below.

**Scheme 1:** Scoring Function of Pfam Domain Sharing [10].

The scoring function 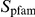 of Pfam domain sharing is simply the number of common domains of the two proteins defined as follows:
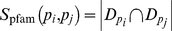
(1)where 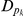 is the set of Pfam domains found in protein 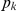.

**Scheme 2:** Scoring Function based on Protein Signature Profiling [13].

The similarity score between a pair of proteins 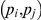 is computed using a binary similarity function between a pair of their signature profiles and is given by
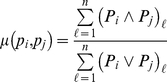
(2)where 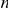 is the number of signatures contained in proteins of a genome of interest and 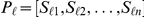 the signature profile of protein 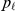, with 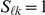, if the signature 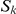 exists in protein 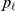 and 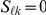 otherwise.

Note that the scheme 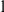 expressed by the equation (1) can be rewritten using Boolean operator ‘and (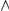)’ as follows:
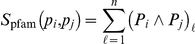
and similarly, the scheme 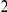 in the equation (2) can also be written using set operators ‘intersection (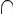)’ and ‘union (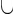)’ as
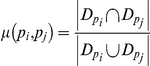
with 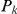 and 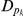 as defined above.

These two schemes just count the number of shared signatures without taking into account the nature of the data and experiments used to derive them. In addition, the limitation of the second scheme can be seen in this small illustration: Let's consider three proteins 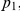 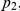 and 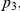 with 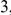 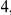 and 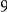 detected signatures, respectively. If we assume that 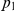 and 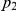 share 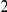 signatures and 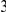 signatures are shared by 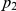 and 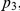 we have: 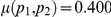 and 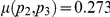. So, 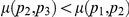, whereas one should expect to have 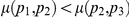 when looking at the number of the common signatures shared by these proteins. In fact, the scoring function as a function of the number of common signatures shared by a pair of proteins, is expected to be increasing. This property does not hold for scoring functions based on protein signature profiling, making this unattractive.

In the case of sequence similarity, the existing scoring schemes rely on the use of the negative logarithm of 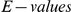 obtained from a sequence similarity tool. As pointed out previously, the problem with these scoring schemes is that initially there is no single fixed 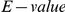 describing where homology ends and non homology begins. This constitutes an impediment to these scoring schemes beyond the fact that they may obviously lead to the singularities caused by the 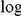 of zeros.

Thus, these schemes are not equipped to capture all the parameters related to the data under consideration and technology used to derive them. In order to overcome these shortcomings, we introduce novel scoring schemes based on the information-theoretic approach, taking into account the nature of the data and technology used and where the user can tune parameters based on their confidence in the data source.

### Scoring Scheme For Protein Family and Domain

Consider two proteins denoted 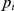 and 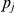, sharing signatures or entries 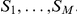 We define the similarity score 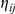 of proteins 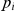 and 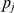 as the minimum number of occurrences of these signatures in proteins 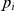 and 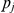, 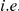,
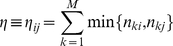
(3)where 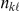 is the number of occurrences of signatures 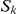 in the protein 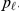


Broadly speaking, the reliability or confidence score increases with the confidence-level of data, which depends on the data source and is torn down by the uncertainty-level of data linked to the dispersion measure 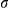. As we are dealing with data from experiments containing a certain level of uncertainty, which propagates into the data, it is natural to use the normal distribution, as these data can be summarized in terms of mean and standard deviation. In fact, in this case this distribution constitutes an attractive approximation as it maximizes information entropy in the data. Thus, we set the confidence-level 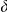 of the similarity score 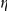 as
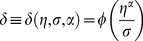
(4)with the function 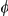 the cumulative probability of the standard Gaussian distribution defined by
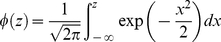
(5)and 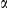 the calibration control parameter, with 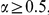 strengthening the impact of the confidence-level for the data under consideration, in which case, 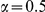 is associated with low confidence data. The training dataset 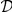 consists of all pairs 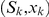, where 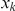 is the number of times the signature 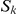 was observed. In order to get rid of observations that lie at abnormal distances from the data, referred to as outliers, it is recommended to use the rectified dataset 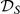, the subset of the training dataset 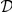 consisting of a data point which falls inside 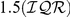, 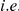,
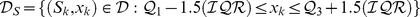
with 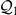 and 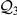, respectively, the 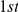 (lower) and 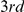 (upper) quartile, and 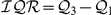 the interquartile range. 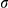 is thus the standard deviation of the rectified dataset, estimated from maximum likelihood and given by
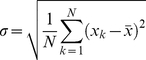
(6)where 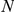 is the number of signatures found in the rectified dataset, and 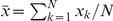, the mean or average of the set.

Given the confidence-level 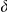 of the similarity score 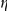 defined in equation (4), the uncertainty measure related to the outcome 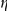 resulting from the data is obtained from the binary entropy function, given by
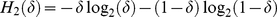
(7)


In fact, the uncertainty measure function 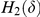 is defined in the interval 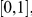 with 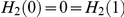 since 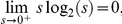 and also 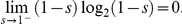 Finally, we set up the capacity of inferring the functional relationship score between two proteins belonging to the same family or sharing common signatures as
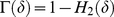
(8)and the reliability or confidence score of the functional relationship between two proteins by
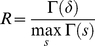
(9)Note that for 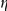 significantly large, 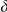 converges to 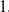 Therefore, the uncertainty measure 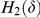 converges to 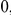 leading to the maximum capacity of inferring the functional relationship of 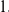 This means that the reliability of a functional relationship between two proteins is given by
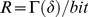
(10)


**Figure 1:**
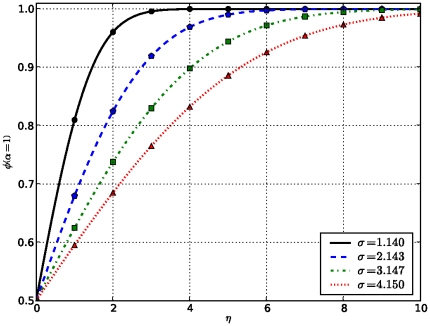
Confidence level variation for 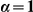. For a fixed calibration control parameter, as the number of shared domains increases, the confidence level also increases with a decrease in the standard deviation 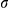.

To illustrate the dependency of this new measure on the data under consideration and the technology used to produce them, we plot the variation of confidence level 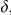 uncertainty 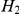 and capacity 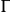 in terms of common domains 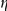 between proteins, for different values of 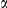, which keeps track of the technology used to produce data and 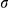 controlling the impact of data under consideration, respectively. These are user-tunable parameters and results are shown in figures 1–4.

**Figure 2 pone-0018607-g002:**
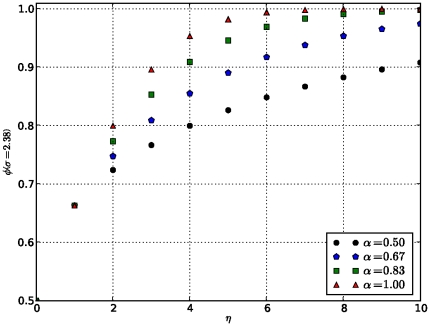
Confidence level variation for 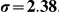 For a fixed standard deviation, as the number of shared domains increases, the confidence level also increases with an increase in the calibration control parameter.

**Figure 3 pone-0018607-g003:**
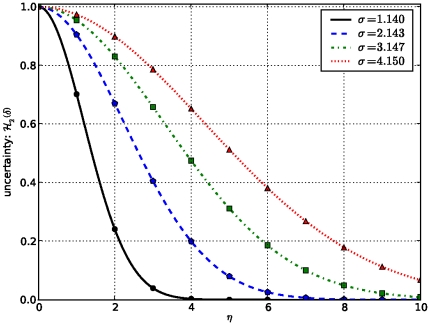
Variation of uncertainty in terms of 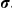 As the number of shared domains increases, the uncertainty composante decreases as the standard deviation 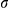 decreases.

**Figure 4:**
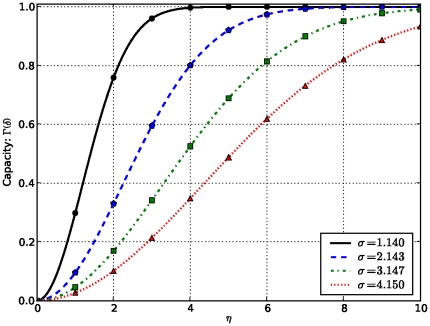
Variation of capacity in terms of 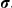 As the number of shared domains increases, the capacity for inferring functional relationships between proteins, and therefore link confidence scores increases as the standard deviation 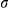 decreases.

These results show that the confidence level 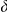 increases as the number of common signatures between the two proteins increases, and that for a higher value of 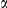, indicating the efficiency level of the technology used to derive data, the confidence level 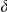 is higher, and so is the reliability or confidence score, due to the fact that in this case the uncertainty component is smaller. Similarly, the impact of data obtained from each technology is taken into account through 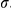 Interestingly, this confidence score formula accommodates the case where no common pattern is found between two proteins in the training dataset, in which case, the confidence score or reliability of a functional relationship is 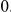 In addition, this scoring scheme takes into account a false positive assignment of any of the common patterns by narrowing down the confidence score of proteins containing only one common signature, depending on the measure of dispersion 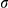 which can provide a hint on the nature of the data under consideration. Indeed, the measure of dispersion 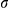 impacts on the confidence score in the sense that if data is far away from the average, in which case 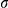 is high, the uncertainty component might be large and significant while calculating the confidence score, thus yielding a lower confidence score. Thus, with knowledge of the data source, the measure of dispersion 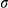 can be penalized by a factor 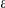 between 0 and 1, in order to reduce the impact of the uncertainty component.

### Scoring Scheme For Protein Sequence Similarity

For a given set of pair-wise homologous sequences, Bastian [19], [ 20] showed that their biological evolution can be formalized by the evolution of their shared amount of information. This is measured by the mutual information in the sense of Hartley [21], [ 22], estimating the information they share due to their common origin and parallel evolution under similar selective pressure. Moreover, this mutual information is proportional to the bit score computed with standard methods in sequence comparisons.

Let 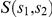 be the bit score alignment of homologous sequences 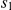 and 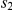, set with its standard units, and 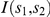 mutual information between these two sequences. We have
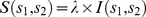
(11)where 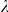 is a constant defining the unity, which depends on the statistical parameter scale 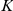 for the search size (http://www.ncbi.nlm.nih.gov/BLAST/tutorial/Altschul-1.html) derived from the scoring matrix and amino acid composition of the sequence [23]. Therefore, generally 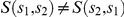 and they are equal only if they have the same scale for the search size. However, the mutual information 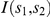 between two sequences 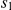 and 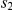 satisfies 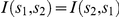 and 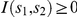 [24].

Equation (11) shows that the mutual information 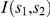 increases with the bit score 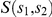, which measures the average information available per position to distinguish an alignment from chance, calculated using relative entropy of target and background distributions [25] as
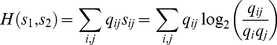
(12)where 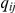 is the “target” residue substitution frequency, the probability of finding a residue 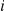 aligned with a residue 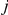 after a certain amount of evolution given that they have both evolved from a common ancestor who had a residue 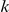 at that position. 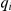 is the probability of occurrence of a residue 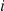 in a collection of sequences, 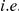, the probability that a residue 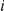 would align by chance based solely on its frequency in a sequence.

Thus, we define the reliability or confidence score 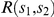 of a functional relationship between two protein sequences 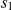 and 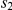 as normalized mutual information calculated [26] as
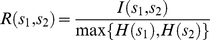
(13)measuring how the protein sequence 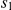 is able to predict the protein sequence 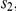 and where 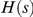 is the relative entropy obtained by aligning a protein sequence 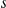 by itself. Indeed, the increase of mutual information with relative entropy yields bias, and this bias is corrected by dividing the mutual information by the maximum entropy of the sequence pair.

Using equation (11), the mutual information 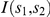 can be computed as follows:
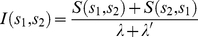
(14)where 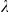 and 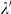 are constants defining unity for 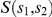 and 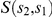, respectively. For a protein sequence 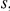 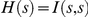, obtained using equation (14) and given by
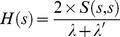
(15)Finally, 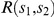 is independent of constants defining unity for 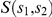 and 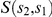, and calculated as
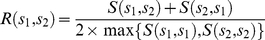
(16)


It is obvious that this scoring scheme relies only on the two protein sequences for which the confidence score is being computed. Two protein sequences whose mutual information of their evolutionary history embedded in their similarity score is 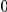, indicates that the two sequences are not similar and so, their confidence score is also 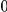. Thus, this scoring scheme accommodates the case where no similarity is found between two protein sequences and the error due to the arbitrary growth of the mutual information between two protein pairs is corrected by the maximum entropy induced.

## Results and Discussion

### MTB Functional Network Derived from Sequence Data

The computation of relationship scores (as described in the methods section) was performed on the whole *Mycobacterium tuberculosis* strain CDC1551 proteome to produce functional links between proteins from homology data, including pair-wise links from sequence similarity and protein family data derived from the InterPro database. Sequence similarity searches were carried out using BLASTP under a BLOSUM62 matrix based on the premise that if the 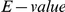 is less than 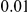, the hit is similar to the query sequence and is likely to be evolutionarily related [27]. Resulting functional link scores are provided in Table S1.

We investigated the general behaviour of the link confidence scores induced from homology datasets. Results are depicted in Table 1 in terms of number and frequency of functional links in a given bin 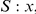 where 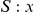 corresponds to link score values ranging between 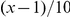 and 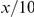 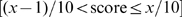.These results indicate that the link confidence scores from protein family data are either low (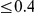) or high (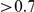). This is due to the calibration control parameter applied to data from the InterPro database, which is 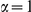 with penalty parameter 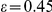, producing either low or high confidence according to the fact that two proteins share only one domain or more than one domain, respectively. Moreover, in most cases, prediction of functional links from sequence similarity matches that of protein family data but at different confidence levels. The link score 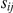 between proteins 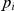 and 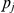 obtained for the combined data is given by
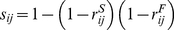
(17)under the assumption of independency, where 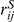 and 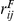 are link confidence scores obtained from sequence similarity and protein family datasets, respectively.

**Table 1:** MTB strain CDC1551 functional links derived from sequence data using our approach, STRING homology scheme for sequence similarity, and using the SFSP approach for protein family and domain sharing.

		Sequence Similarity		Protein Family and Domain			
Confidence	Bins	Our Approach	STRING scheme	Our Approach	SFSP-Under	SFSP-Aver	SFSP-Over
Low	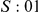	4321	0	0	33240	0	0
	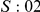	3001	0	0	4365	0	0
	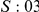	1206	0	0	814	0	0
	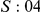	606	44	20915	172	27494	0
Medium	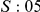	424	263	0	6	6	6
	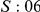	215	140	0	41	5746	0
	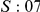	96	99	0	45	1394	0
High	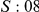	31	57	7847	0	3906	0
	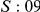	21	58	0	18	155	45
	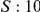	25	52	9945	6	6	38656
Medium-High Total:		812	669	17792	116	11213	38707
Overall Total :		9946	713	38707	38707	38707	38707

Number of Interactions per Source and Link Score shown separately by bin.

### Evaluating the Scoring Scheme

We compared our approach for scoring functional interactions inferred from sequence similarity to the STRING homology scoring scheme. STRING is a database of known and predicted protein-protein associations for a large number of organisms derived from high-throughput experimental data, the mining of databases and literature, and from predictions based on genomic analysis. For this assessment we used only their links derived from homology data, which uses a scoring scheme based on E-values obtained from the Smith-Waterman algorithm with a reasonably strict cut-off score to ensure high quality matches [28]. We also compared our approach for scoring functional interactions from protein family and domain to the scoring scheme for protein signature profiling (SFSP).

The STRING scheme classifies its functional link confidence scores into three different categories, low, medium and high confidence, with corresponding scores less than 0.4, between 0.4 and 0.7, and greater than 0.7, respectively [11]. These scores measure our confidence in pair-wise functional interactions in the networks produced. Even though sequence data are initially accurate, computational tools used to produce sequence similarity data may introduce noise due to certain unpredictable factors, such as arbitrary increases of bit score or over-estimation of similarity patterns between sequences. In order to take into account these uncertainties in sequence similarity data while ensuring the accuracy of functional interactions produced, one can set a cut-off score above which a given interaction is more likely to occur. Therefore, the comparison was performed in terms of functional classification accuracy for links with a medium confidence level and upwards (link score greater than 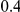). The number of associations predicted in different MTB functional networks produced using different approaches are shown separately in Table 1 for each approach and confidence ranging from low to high.

**Figure 5:**
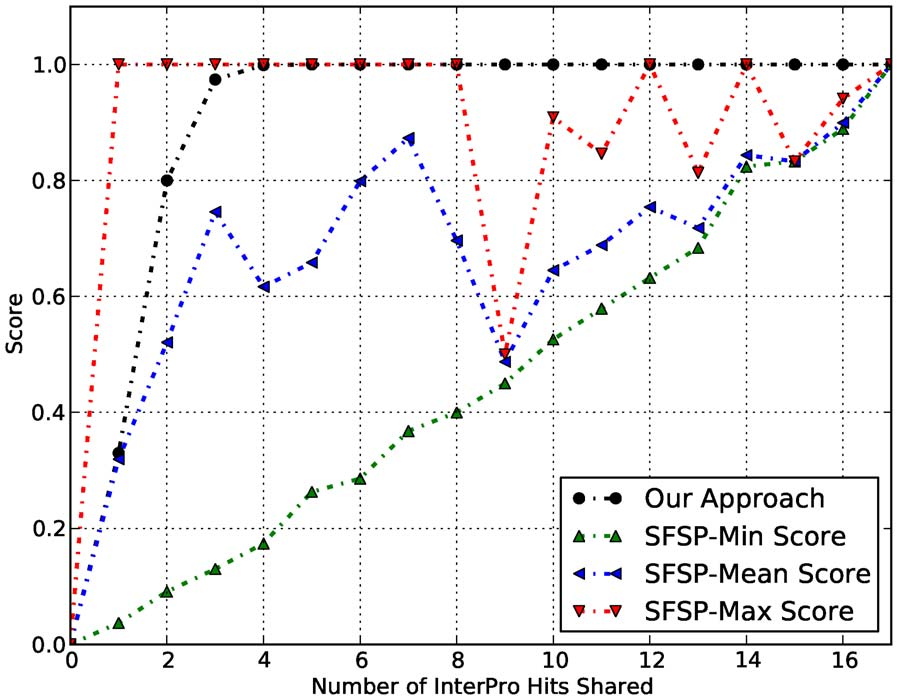
Variation of Scores in the Protein Signature Profiling (SFSP) based approach compared to our approach. Change in Protein Signature Profiling Score minimum, mean and maximum and our approach when varying the number of shared domains between proteins.

The SFSP as defined by equation (2) may produce several link scores for the same number of shared domains, we have considered the maximum score when over-estimating, their minimum when underestimating and their average score, referred to as SFSP-Max, SFSP-Under and SFSP-Mean, respectively. We plot the scores obtained using our approach and these from SFSP, and results are shown in figure 5. As pointed out previously, the scoring function should be increasing since our confidence level increases with the number of common signatures shared between pair-wise proteins. These results show that only SFSP-Under estimation provides the increasing scoring function but unfortunately it yields a poor coverage and for this reason it is not considered for further performance evaluation. The scoring scheme developed here produces an increasing scoring function and provides a better trade-off between SFSP-Max and SFSP-Mean. Considering the confidence score cut-off applied, the configuration of the network produced from SFSP-Max estimation is the same as that derived using the scheme based on the scoring function of domain sharing described by equation (1).

### Statistical significance of Functional Interactions Derived

We evaluated the statistical significance and biological relevance of the functional interactions inferred using our scoring approach in terms of functional classification coherence. To measure this, an interaction between two proteins is said to be significant or correct if these proteins belong to the same functional class.

The functional classes were extracted from Tuberculist (http://genolist.pasteur.fr/Tuberculist), and the repartition of interacting proteins in the functional network per functional class or category for different configurations is shown in Table 2. The evaluation was done using a sub-network generated by each protein in the functional network, consisting of functional interactions between a protein under consideration and its direct neighbours, referred to as a P-subgraph. The proteins in the unknown functional class were excluded from the evaluation.

**Table 2:** Distribution of MTB strain CDC1551 proteins per functional class.

		Sequence Similarity		Protein Family and Domain			
	Functional Class	Our Approach	STRING Scheme	Our Approach	SFSP-Under	SFSP-Aver	SFSP-Over
1	Virulence, detoxification and adaptation	34	33	89	0	82	143
2	Lipid Metabolism	47	97	190	19	133	222
3	Information Pathways	12	21	148	2	125	183
4	Cell-wall and Cell Process	82	101	236	2	181	355
5	Stable RNAs	-	-	-	-	-	-
6	Insertion Sequences and Phages	32	2	42	0	30	55
7	PE/PPE/PGRS Proteins	89	43	59	0	57	142
8	Intermediary Metabolism and Respiration	65	174	603	1	508	759
9	Protein of Unknown Function	77	77	287	0	222	555
10	Regulatory Proteins	17	14	148	0	145	165
	Total	455	562	1802	24	1483	2579

Number of proteins per functional class in the functional networks produced using our approach and the STRING homology scheme, and using the SFSP approach for protein family and domain sharing.

To assess functional category coherence of functional interactions derived from a random model, we compute the P-value for each P-subgraph defined as the probability that the P-subgraph under consideration occurs by chance or is comprised of randomly drawn interactions. The hypergeometric distribution, which yields the probability of observing at least 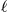 interactions between proteins from a given P-subgraph of size 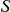 by chance among 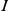 interactions of the same type in the entire functional network considered to be a background distribution, is used to model the P-value [14] given by
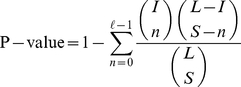
(18)where 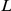 is the size of the functional network, 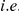, the number of functional links in the network, with all the proteins in the unknown class removed.

**Figure 6:**
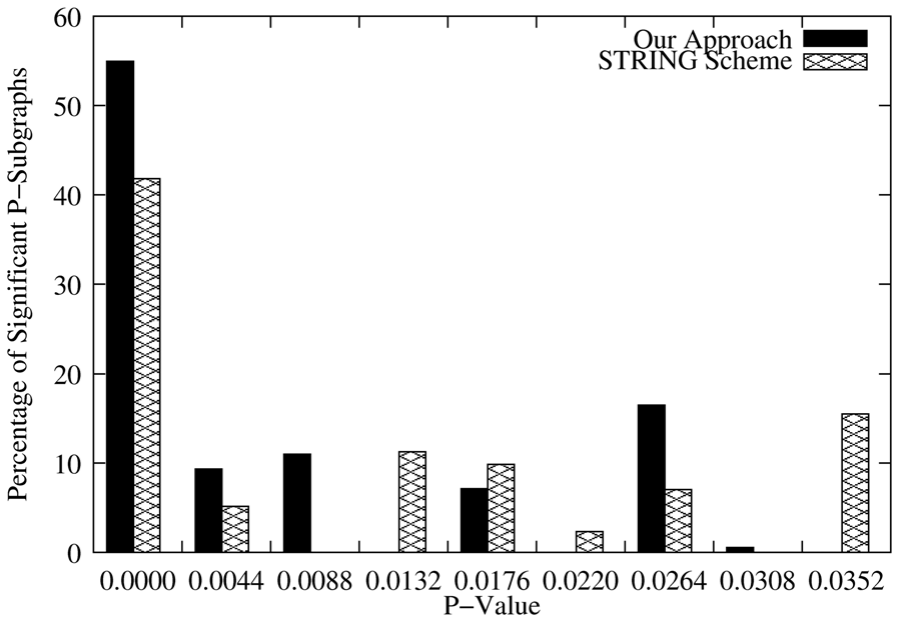
Significance of functional interactions derived using our approach and the STRING scheme. At each significance level 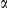 in these graphs, we counted all relevant predicted associations for the two approaches and computed the percentage. Each 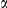 corresponds to the number of associations with p-value 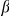 and 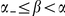, where 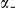 is the significance level just before 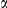 in the plot.

**Figure 7:**
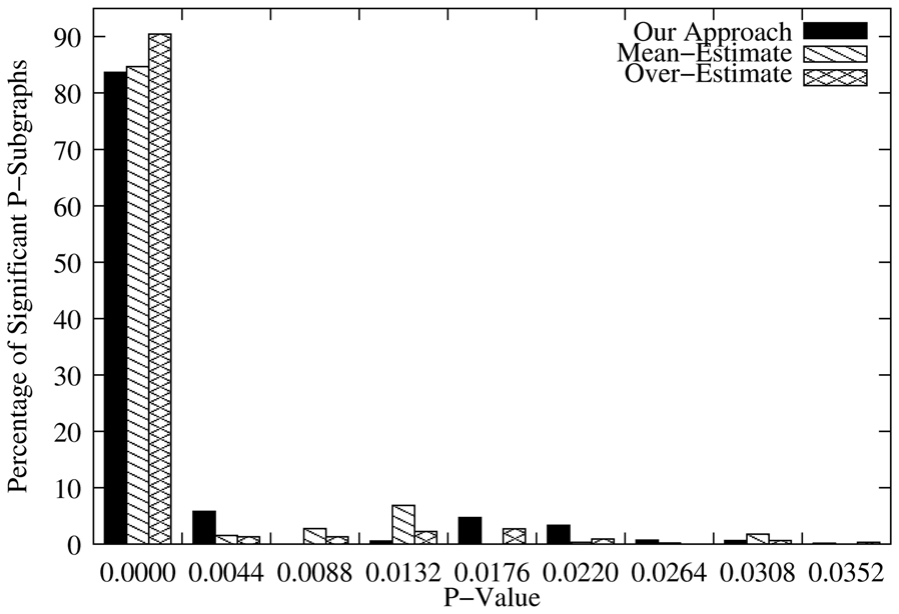
Significance of functional interactions derived using our approach and SFSP approach. At each significance level 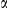 in these graphs, we counted all relevant predicted associations for the two approaches and computed the percentage. Each 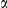 corresponds to the number of associations with p-value 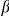 and 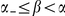, where 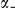 is the significance level just before 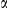 in the plot.

We assessed functional category coherence of functional interactions derived using our approach and STRING homology data for sequence similarity, as well as those inferred using our scheme for protein family and domain, and those obtained using SFSP-Mean and SFSP-Max estimation. Results displayed in figures 6 and 7 show that the functional interactions induced have a very low probability of occurring by chance. Note that this statistical test against a random distribution aims at checking if a given P-subgraph in the functional network consists of randomly grouped proteins. These figures show that using a significance level of 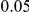 as the optimal threshold, more P-subgraphs derived using our approach are statistically significant than those obtained from the STRING homology scoring and provides roughly equal statistically significant percentage of P-subgraphs with SFSP-Mean and SFSP-Max schemes. A total of 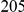 out of 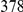, representing 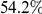 of P-subgraphs in our network are significant compared to 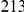 out of 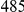 representing 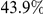 of P-subgraphs for the STRING scoring system for sequence similarity. For SFSP scheme for protein family and domain, A total of 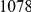 out of 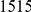 representing 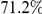 of P-subgraphs in our network are significant compared to 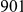 out of 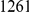 representing 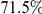 of P-subgraphs for SFSP-Mean and to 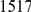 out of 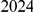 representing 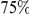 for SFSP-Max.

### Effectiveness of The Novel Scoring Scheme

To evaluate the classification power of the new scoring scheme, we used the modified Receiver Operator Characteristic (ROC) curve analysis that measures the number of true positive (TP) predictions (number of functional interactions correctly identified) against the number of false positive (FP) (number of functional interactions incorrectly identified) [29], in which case the area under the ROC curve (AUC) is used as a measure of discriminative power. The larger the upper AUC value (the portion between the curve and the line TP  =  FP), the more powerful the scheme is.

**Figure 8:**
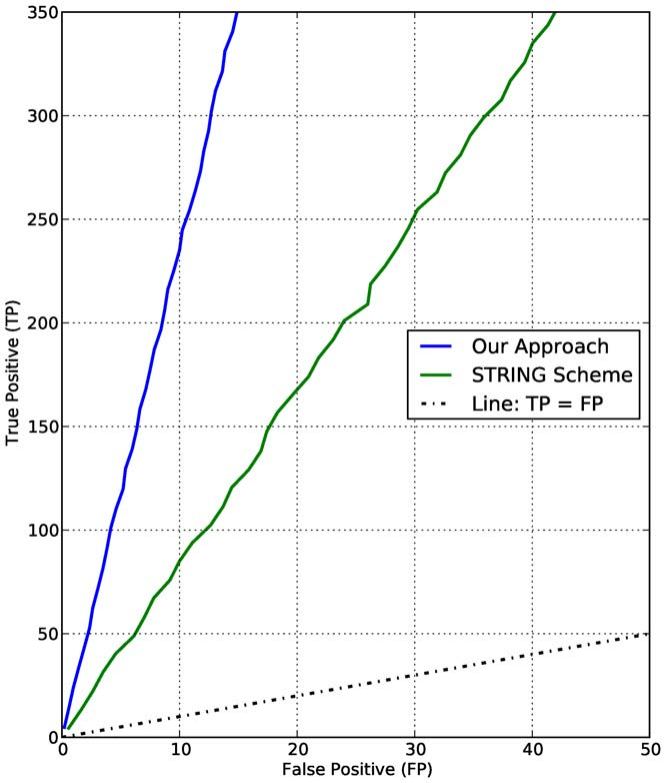
Modified ROC curves for functional interactions. Number of incorrect functional interactions (false positives) versus number of correct functional interactions (true positives) in the MTB strain CDC1551 functional networks produced by our approach and the STRING homology network for sequence similarity.

**Figure 9:**
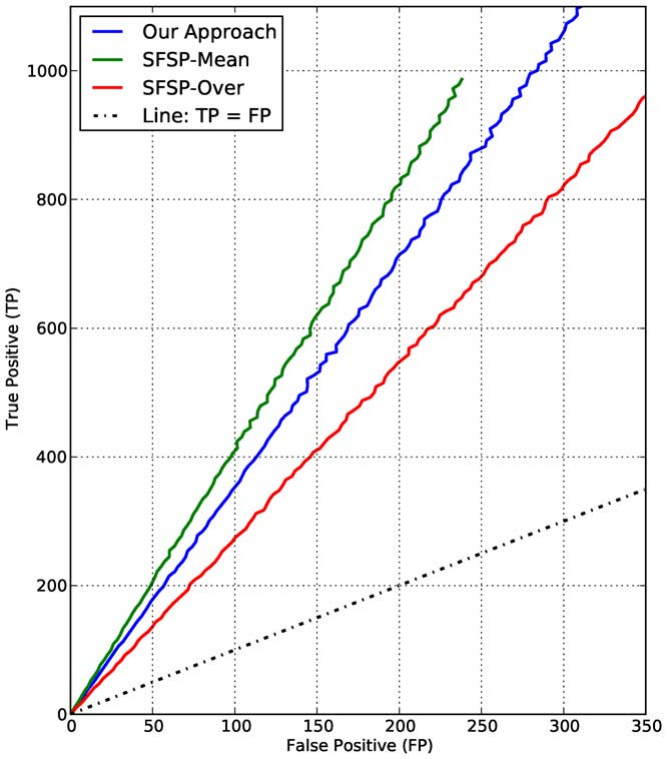
Modified ROC curves for functional interactions. Number of incorrect functional interactions (false positives) versus number of correct functional interactions (true positives) in the MTB strain CDC1551 functional networks produced by our approach and the SFSP scheme for protein family and domain.

For a given number of P-subgraphs ranging from 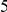 to 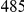, we randomly generated 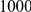 independent samples and compute the average number of correct and incorrect predicted interactions expected to be normally distributed from the central limit theorem. Thus, we perform modified ROC analyses for the two scoring approaches, and results are shown in figure 8 for sequence similarity. These results indicate that our approach outperforms the STRING scheme, respectively, with an average of 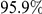 and 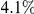 of functional interactions correctly and incorrectly identified out of 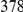 P-subgraphs, compared to the STRING scheme, which provides an average of 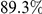 and 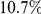 of functional interactions correctly and incorrectly identified, respectively, out of 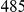 P-subgraphs. This shows not only that it is not sufficient to ensure high quality matches [28] by just applying a reasonably strict cut-off score when using the Smith-Waterman algorithm, but also this practice may lead to a poor coverage. Results in figure 9 indicate that our method performs comparably to the SFSP-Max and SFSP-Mean schemes, and provides a better trade-off between over-estimating and averaging scores for SFSP schemes in terms of precision and coverage. Our approach provides an average of 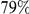 and 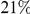 of functional interactions correctly and incorrectly, respectively, identified out of 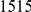 P-subgraphs. SFSP-Mean yields an average of 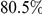 and 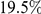 of functional interactions correctly and incorrectly identified, respectively, out of 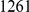 P-subgraphs while SFSP-Max produces an average of 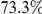 and 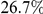 of functional interactions correctly and incorrectly identified, respectively, out of 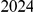 P-subgraphs. Apart from the general limitation common to scoring schemes inferred from signature profiling based approaches, SFSP-Max produces a poor precision. This poor performance is due to the fact that when over-estimating it includes all false positives and our approach corrects this, providing an improved precision and coverage.

### General Analysis of the Structure of the Functional Network Produced

**Figure 10:**
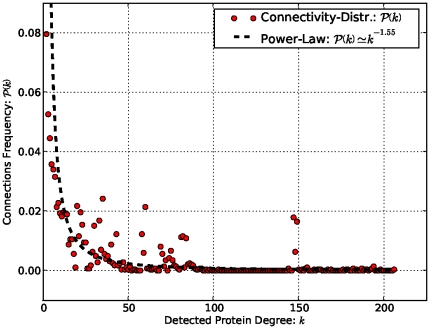
Power law property of MTB strain CDC1551 functional network obtained from sequence data. Connectivity distribution of detected functional links 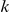 per protein, plotted as a function of frequency 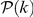.

We performed a general analysis of the homology-based functional network produced by integrating into a single network all functional interactions inferred from sequence similarity and protein family and domain data using our scheme. The number of functional links in the combined network, which contains a total of 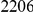 proteins (nodes), is given in Table 3. The results in figure 10 show that this network exhibits scale-free topology, 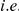, the degree distribution of proteins approximates a power law 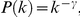 with the degree exponent 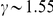. We analyzed the general behavior of this network by finding the number of cliques and the distribution of hubs. Here protein hubs are described as “single points of failure” able to disconnect the network. This functional network contains 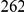 clusters, or cliques, with 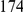 hubs and with the biggest cluster containing 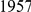 gene products.

**Table 3:** MTB strain CDC1551 functional links derived from sequence data using our approach.

		Interactions from	Interactions From Protein	
Confidence	Bins	Sequence Similarity	Family (InterPro data)	Combined Interactions
Low	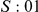	4321	0	206
	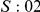	3001	0	125
	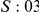	1206	0	62
	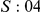	606	20915	18381
Medium	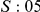	424	0	1634
	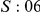	215	0	605
	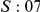	96	0	262
High	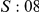	31	7847	6998
	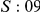	21	0	855
	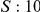	25	9945	10022
Medium-High Total:		812	17792	20376
Overall Total :		9946	38707	39150

Number of Interactions per Source and Link Score shown separately by bin.

### Predicting Protein Functional Class

Several approaches have been proposed for predicting protein functions from functional networks and are mainly classified into two categories, namely global network topology and local neighborhood based approaches. Global network topology based approaches use global optimization [30]–[32] or probabilistic methods [33]–[36] or machine learning [37]–[39] to improve the prediction accuracy using the global structure of the network under consideration. Unfortunately, these approaches raise a scalability issue which might not be proportional to the improvement in predictions compared to most straight forward approaches, which rely only on local neighborhood [40] of uncharacterized proteins.

In the case of local neighborhood based approaches, known as ‘Guilt-by-Association’ or ‘Majority Voting’ or ‘Neighbor Counting’ [41], direct interacting neighbors of proteins are used to predict protein functions. However, the biggest limitation of approaches relying on the direct neighbors of the protein under consideration is that they are unable to characterize proteins whose direct interacting neighbors are all uncharacterized, thus impacting negatively on annotation coverage. Investigating the relation between interacting neighbors of a given protein using network topology, Chua et al. [8], [42] show that in many cases, a protein shares functional similarity with level-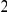 neighbors (2 branch-lengths away) and proposed a functional similarity weight (FS-Weight) method for predicting protein functions from protein interaction data. Here, we analyze the performance of using direct interacting neighbors and second level interacting neighbors. The second level interacting neighbors were used when we were unable to use direct interacting neighbors, in order to improve coverage.

The functional network produced from sequence data was used to predict, where possible, the functional class of proteins in the Tuberculist unknown functional class using a local neighborhood based approach. Through this, a new functional class is assigned to an unknown protein based on the functional class frequently occurring among its direct interacting neighbors. In this case, the score of a given functional class 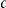 for a protein 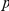 is given by the frequency 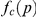 of occurrence of functional class 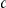 among direct neighbors of 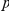, and calculated as follows:
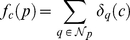
(19)where 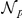 refers to the set of direct interacting partners of protein 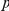, and 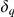 is the 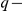
function indicator given by
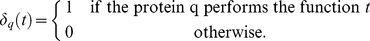



Since the objective is to assign to an unknown protein only one functional class, we make use of global network information, and the prediction of a given protein functional class is based on an over represented functional class found amongst its direct neighbors. The functional class with the largest chi-squared score is assigned to the protein. The chi-square score of functional class 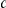 for protein 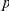 [43] is given by
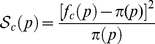
(20)where 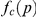 is defined in equation (19) and 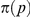 is the global expected number of proteins belonging to the functional class 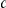, given by 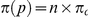, with 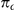 that of proteins belonging to the class 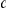 among all the proteins in the functional network under consideration and 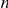 the order of the functional network, 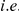, number of proteins in the network.

**Figure 11:**
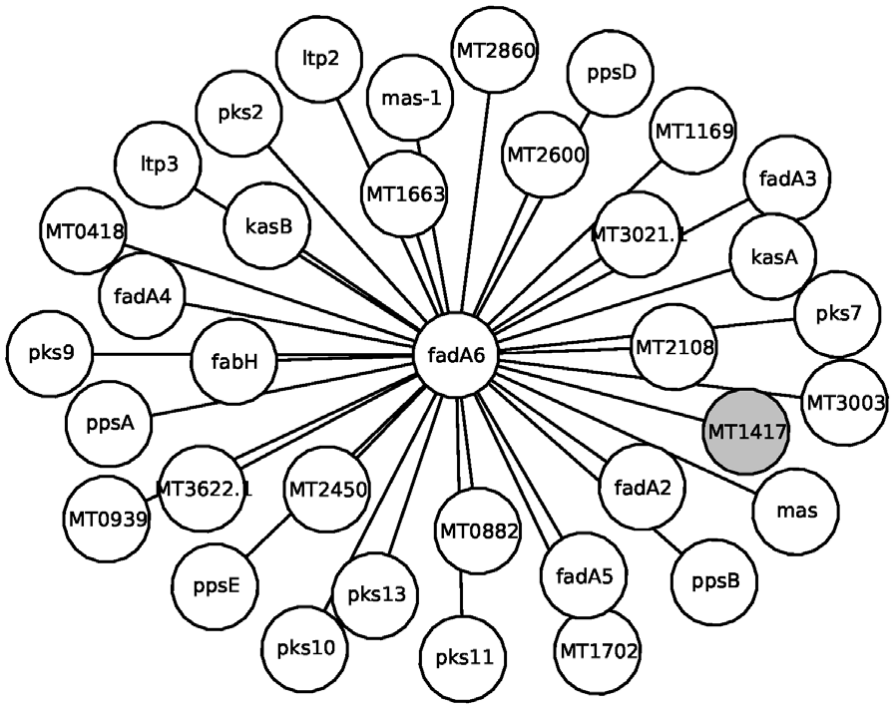
Illustration of Guilt-By-Association using level-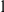 interacting neighbors for protein classification. P-subgraph showing the direct interacting partners of protein ‘FAdA6’ (in the center shown in white). Proteins in white are involved in lipid metabolism, while the gray nodes are of the unknown class.

**Figure 12:**
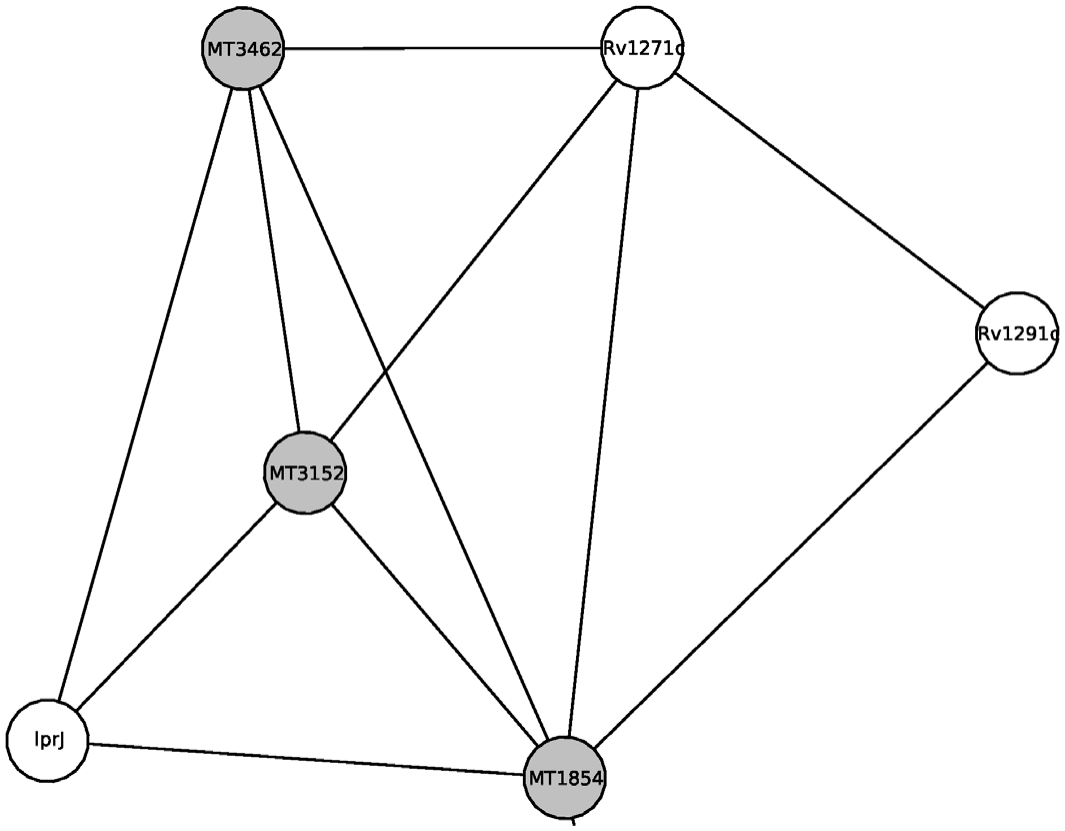
Illustration of Guilt-By-Association using level-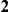 interacting neighbors for protein classification. Graph depicting level-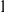 and level-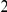 interacting partners of protein ‘lprJ’. Proteins in white are involved in lipid metabolism and those shown in gray are of unknown class.

**Figure 13:**
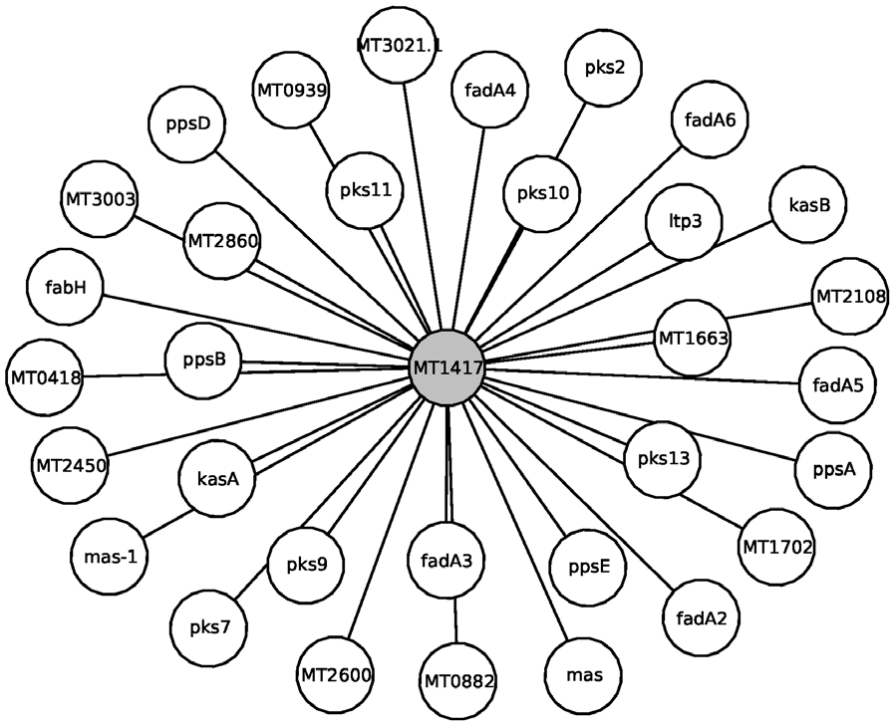
Illustration of protein functional classification inferrence. P-subgraph showing the direct interacting partners of protein ‘M1417’ (gray node in the center) of unknown class. Proteins in white are involved in lipid metabolism.

As an illustration, protein ‘fadA6’ (MT3660 or Rv3557c), named Acetyltransferase FADA6 (UniProt accession P96834), which is involved in lipid metabolism (figure 11), is functionally linked to proteins annotated to the lipid metabolism class. This means that if we assumed that the protein ‘fadA6’ was not classified then it is likely that ‘fadA6’ would have been annotated to the lipid metabolism class. Similarly, protein ‘lprJ’ (MT1729 or Rv1690), named lipoprotein LPRJ (O33192), is also known to be involved in lipid metabolism (figure 12). All its direct interacting partners are of the unknown class, in which case if the class of ‘lprJ’ was not known, the use of level-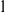 neighbors would fail to classify this protein. However, using the level-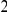 neighbors would successfully classify this protein. Finally, figure 13 shows protein MT1417 (Rv1372, Q7D8I1), which is of unknown class in Tuberculist, but suggested by UniProt to belong to the chalcone/stilbene synthase family known to be involved in lipid metabolism. The prediction method annotates this protein to lipid metabolism, thus confirming the suspicion.

**Figure 14:**
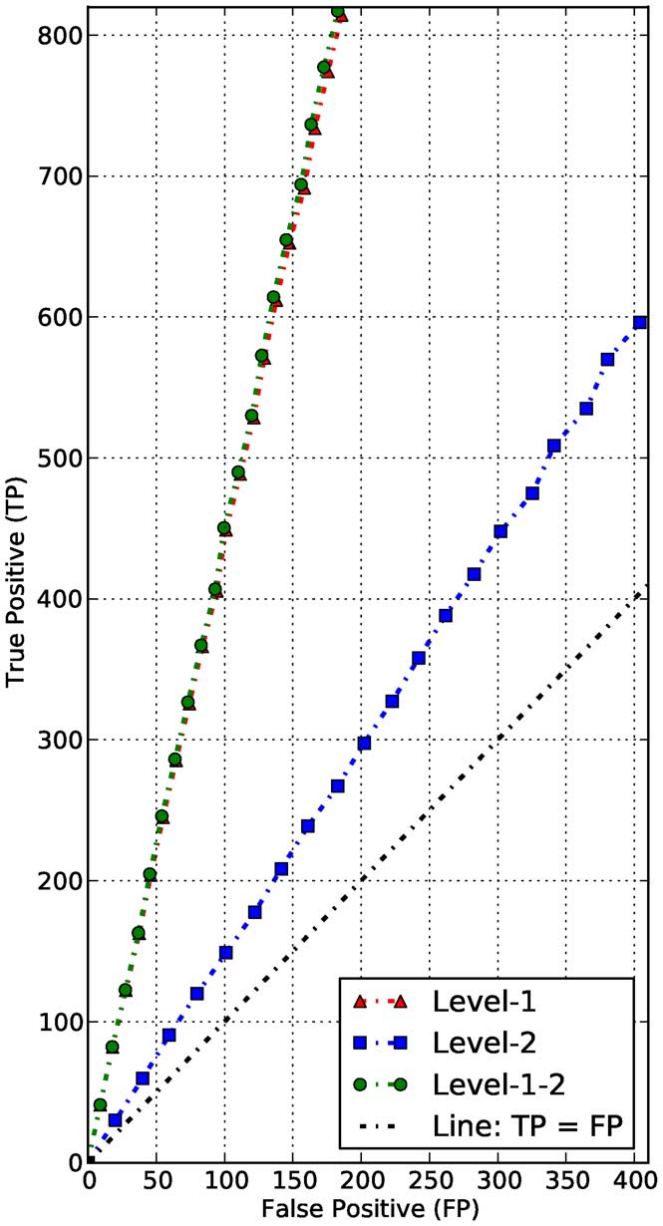
Performance evaluation of classification prediction approaches. Number of proteins incorrectly classified (false positives) versus number of proteins correctly classified (true positives) using level-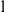, level-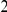, and combined level-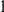 and level-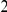 interacting partners to improve coverage.

Once again, the classification performance of these approaches can be evaluated with modified ROC curve analyses. We used leave-one-out cross-validation to evaluate the efficiency of these prediction approaches at computing the number of proteins correctly classified and those incorrectly classified. Note that when using the level-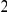 interacting neighbors to classify a protein, the instance of each protein is counted, 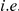, if a given level-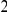 neighbor interacts with different direct interacting neighbors, it will be counted twice. In order to compare the effectiveness of these approaches, we combined their related modified ROC curves and results are shown in figure 14. These results indicate that while the level 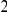 interacting partners may be used to improve the coverage, they contain many false positives impacting negatively on the precision. Combining level 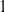 and level 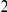 interacting partners slightly improves precision and coverage. These two measures of protein classification quality are computed as follows:
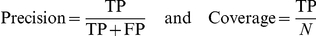
where TP (true positive) is the number of proteins correctly classified, 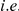, number of proteins for which the actual classification is the same as the one predicted, FP (false positive) is the number of proteins for which the classification is different to the one predicted, and 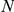 is the total number of classified proteins in the functional network. Thus, the precision measures the proportion of proteins with correct classifications among all proteins classified, and coverage measures the proportion of proteins correctly classified among the proteins in the functional network. The use of level-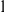 neighbors provides a precision of 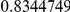 with a coverage of 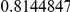, while level-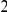 neighbors produces a precision of 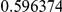 with a coverage of 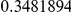. Combining level-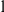 and 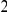 neighbors yields a precision of 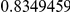 with a coverage of 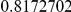. This is only a slight improvement over using level-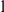 neighbors only, but the illustration for LPRJ above shows the value in using both.

### Conclusions

We have developed novel information-theoretic based schemes for calculating the link confidence scores or link reliability for homology data, 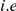, data from protein family and sequence similarity. These convert the amount of biological content shared between proteins into confidence scores of their functional relationships. The methods could be used for a clustering analysis but here they are used for functional network generation.

We applied these schemes to the genome of *Mycobacterium tuberculosis* strain CDC1551 to produce a protein-protein functional network. Results showed that the novel scheme is efficient and effective compared to the existing schemes and can be used to improve functional networks inferred from sequence data in terms of precision and coverage.

We analyzed the global behaviour of the network obtained from the new scoring schemes. Furthermore, the functional network produced was used to classify proteins in the unknown class using a local neighborhood based approach extended to level-2 protein neighbors in order to improve genomic coverage.

Currently, we are integrating into a single protein-protein functional network, all pair-wise functional interactions obtained from different data sources, including genetic interactions, and functional genomics data, in order to predict functions, where possible, of uncharacterized proteins in the genome and to study the biology of the organism.

## Supporting Information

Table S1 **# scores of functional interactions derived from sequence data.**
(XLS)

## References

[1] Baldi P, Brunak S (2001). BIOINFORMATICS: The Machine Learning Approach,. Massachusetts Institute of Technology.

[2] Hunter S, Apweiler R, Attwood TK, Bairoch A, Bateman A (2009). InterPro: the integrative protein signature database,. Nucleic Acids Research.

[3] Altschul SF, Gish W, Miller W, Myers EW, Lipman DJ (1990). A basic local alignment search tool,. Journal of Molecular Biolology.

[4] Altschul SF, Madden TL, Shaffer AA, Zhang J, Zhang Z (1997). Gapped BLAST and PSI-BLAST: a new generation of protein database search programs,. Nuceic Acids Research.

[5] UniProt Consortium (2007). The Universal protein resources,. Nucleic Acid Research.

[6] Mulder NJ, Apweiler R, Attwood TK, Bairoch A, Bateman A (2007). New Development in InterPro Database,. Nucleic Acid Research.

[7] Mulder NJ, Apweiler R, Attwood TK, Bairoch A, Bateman A InterPro, progress and status in 2005,. Nucleic Acids Research.

[8] Chua HN, Sung WK, Wong L (2006). Exploiting indirect neighbours and topological weight to predict protein function from protein-protein interactions,. Bioinformatic.

[9] Myers CL, Troyanskaya OG (2007). Context data integration and prediction of biological networks,. Bioinformatics.

[10] Chua HN, Sung WK, Wong L (2007). An efficient strategy for extensive integration of diverse biological data for protein function prediction,. Bioinformatics.

[11] von Mering C, Jensen LJ, Snel B, Hooper SD, Krupp M (2005). STRING: known and predicted protein-protein associations, integrated and transferred across organisms,. Nucleic Acids Research.

[12] Devos D, Valencia A (2000). Practical limits of function prediction,. PROTEINS: Structure, Function, and Genetics.

[13] Mahdavi MA, Lin Y-H (2007). Prediction of Protein-Protein Interactions Using Protein Signature Profiling,. Genomics, Proteomics & Bioinformatics.

[14] Mao X, Cai T, Olyarchuk JG, Wei L (2005). Automated genome annotation and pathway identification using the KEGG Orthology (KO) as a controlled vocabulary,. Bioinformatics.

[15] Yellaboina S, Goyal K, Mande SC (2007). Inferring genome-wide functional linkages in E. coli by combining improved genome context methods: Comparison with high-throughput experimental data,. Genome Research.

[16] Raman K, Yeturu K, Chandra N (2008). targetTB: A target identification pipeline for Mycobacterium tuberculosis through an interactome, reactome and genome-scale structure analysis,. BMC Systems Biology.

[17] Krawczyk J, Kohl TA, Goesmann A, Kalinowski J, Baumbach J (2009). From Corynebacterium glutamicum to Mycobacterium tuberculosis-towards transfers of gene regulatory network and integrated data analyses with MycoRegNet,. Nucleic Acid Research.

[18] Jensen LJ, Kuhn M, Stark M, Chaffron S, Creevey C (2008). STRING 8-a global view on proteins and their functional interactions in 630 organisms,. Nucleic Acids Research.

[19] Bastian O, Ortet P, Roy S, Maréchal E (2005). A configuration space of homologous proteins conserving mutual information and allowing a phylogeny inference based on pair-wise Z-score probabilities,. BMC Bioinformatics.

[20] Bastian O, Maréchal E (2008). Evolution of Biological sequences implies an extrema value distribution of type I for both global and local pair-wise alignments scores,. BMC Bioinformatics.

[21] Hartley RVL (1928). Transmission of Information,. The Bell System Technical Journal.

[22] Shannon CE (1948). A Mathematical Theory of Communication,. The Bell System Technical Journal.

[23] Pearson WR Protein sequence comparison and Protein evolution,. Tutorial-ISBM2000.

[24] Mackay JCD (2004). Information Theory, Inference, and Learning algorithms,. Cambridge University Press.

[25] Altschul SF (1991). Amino acid substitution matrices from an information theoretic perspective,. J. Mol. Biol..

[26] Li M, Chen X, Li X, Ma B, Vitányi MBP (2004). The Similarity Metric,. IEEE transactions on Information Theory.

[27] Subramanian G, Koonin EV, Aravind L (2000). Comparative Genome Analysis of the Pathogenic Spirochetes Borrelia burgdorferi and Treponema pallidum,. Infection and Immunity.

[28] von Mering C, Jensen LJ, Kuhn M, Chaffron S, Doerks T (2007). STRING 7-recent developments in the integration and prediction of protein interactions,. Nucleic Acids Res..

[29] Aaron PG, Sonia ML, William AB, Lawrence EH, Debra SG (2008). Improving protein function prediction methods with integrated literature data,. BMC Bioinformatics.

[30] Vazquez A, Flammini A, Maritan A, Vespignani A (2003). Global protein function prediction from protein-protein interaction networks,. Nature Biotechnology.

[31] Tsuda K, Shin H, Schölkopf B (2005). Fast protein classification with multiple networks,. Bioinformatics.

[32] Nabieva E, Jim K, Agarwal A, Chazelle B, Singh M (2005). Whole-proteome prediction of protein function via graph-theoretic analysis of interaction maps,. Bioinformatics.

[33] Troyanskaya OG, Dolinski K, Owen AB, Altman RB, Botstein D (2003). A Bayesian framework for combining heterogeneous data sources for gene function prediction (in Saccharomyces cerevisiae),. PNAS.

[34] Deng M, Chen T, Sun F (2004). An Integrated Probabilistic Model for Functional Prediction of Proteins,. Journal of Computational Biology.

[35] Letovsky S, Kasif S (2003). Predicting protein function from protein/protein interaction data: a probabilistic approach,. Bioinformatics.

[36] Cho Y-R, Shi L, Ramanathan M, Zhang A (2008). A probabilistic framework to predict protein function from interaction data integrated with semantic knowledge,. BMC Bioinformatics.

[37] Lanckriet GRG, Deng M, Cristianini N, Jordan MI, Noble WS (2004). Kernel-Based Data Fusion and Its Application to Protein Function Prediction in Yeast,. Pacific Symposium on Biocomputing.

[38] Chen Y, Xu D (2004). Global protein function annotation through mining genome-scale data in yeast Saccharomyces cerevisiae,. Nucleic Acids Research.

[39] Xiong J, Rayner S, Luo K, Li Y, Chen S (2006). Genome wide prediction of protein function via a generic knowledge discovery approach based on evidence integration,. BMC Bioinformatics.

[40] Murali TM, Wu CJ, Kasif S (2006). The art of gene function prediction,. Nature Biotechnology.

[41] Schwikowski B, Uetz P, Fields S (2000). A network of protein-protein interactions in yeast,. Nature Biotechnology.

[42] Chua HN, Sung WK, Wong L (2007). Using Indirect Protein Interactions for the Prediction of Gene Ontology Functions,. BMC Bioinformatics.

[43] Deng M, Sun F, Chen T (2003). Assessment of the reliability of protein-protein interactions and protein function prediction,. Pacific Symposium on Biocomputing.

